# Is the construct stability of the acetabular cup affected by the acetabular screw configuration in bone defect models?

**DOI:** 10.1186/s13018-023-03845-y

**Published:** 2023-05-12

**Authors:** Ho-Jin Nam, Young-Wook Lim, Woo-Lam Jo, Ji Hoon Bahk, Soon-Yong Kwon, Hyung Chul Park, Saad Mohammed AlShammari

**Affiliations:** 1 Asan Catholic Orthopedic Clinic, 13 Baebang-ro, Baebang-eup, Asan-si, Chungcheongnam-do 31482 South Korea; 2 Department of Orthopaedic Surgery, School of Medicine, Seoul St. Mary’s Hospital, The Catholic University of Korea, 222, Banpo-daero, Seocho-gu, 06591 Seoul Korea; 3 Department of Orthopaedic Surgery, School of Medicine, Bucheon St. Mary’s Hospital, The Catholic University of Korea, 327, Sosa-ro, Bucheon-si, 14647 Gyeonggi-do Korea; 4 Department of Orthopaedic Surgery, King Abdulaziz Air Base Hospital, Ministry of Defence, 75M2+R7R, King Abdel Aziz Air Base, Dhahran, 34641 Saudi Arabia

**Keywords:** Construct stability, Revision hip surgery, Torsional strength, Lever-out strength

## Abstract

**Background:**

In revision surgery with significant segmental acetabular defects, adequate implant selection and fixation methods are critical in determining successful bony ingrowth. Commercially available total hip prosthesis manufacturers generally offer additional multi-hole options of acetabular shells with identical designs for use in revision THAs where screw holes configurations vary from product to product. This study aims to compare the mechanical stability of the two types of acetabular screw constructs for the fixation of acetabular components: spread-out and pelvic brim-focused configurations.

**Methods:**

We prepared 40 synthetic bone models of the male pelvis. In half of the samples with acetabular defects, identical curvilinear bone defects were manually created using an oscillating electrical saw. On the right side, multi-hole-cups in which the direction of the screw holes are centered on the pelvic brim (brim-focused) and, on the left side, multi-hole-cups with the direction of the screw hole spread throughout the acetabulum (spread-out) were implanted into the pelvic synthetic bones. Coronal lever-out and axial torsion tests were performed with a testing machine, measuring load versus displacement.

**Results:**

The average torsional strengths were significantly higher in the spread-out group over the brim-focused group regardless of the presence of the segmental defect of the acetabulum (*p* < 0.001). But for the lever-out strength, the spread-out group exhibited significantly higher average strength over the brim-focused group for the intact acetabulum (*p* = 0.004), whereas the results were reversed in the brim-focused group when the defects were generated (*p* < 0.001). The presence of acetabular defects reduced the average torsional strengths of the two groups by 68.66% versus 70.86%. In comparison, the decrease in the average lever-out strength was less significant for the brim-focused group than the spread-out group (19.87% vs. 34.25%) (*p* < 0.001).

**Conclusion:**

Constructs of multi-hole acetabular cups with the spread-out screw holes configuration exhibited statistically better axial torsional strength and coronal lever-out strength. With the presence of posterior segmental bone defects, the spread-out constructs demonstrated significantly better tolerance to axial torsional strength. Still, they exhibited inverted results of higher lever-out strength in the pelvic brim-focused constructs.

## Introduction

Successful acetabular cup fixation in cementless total hip arthroplasty (THA) is achieved by the initial mechanical stability of the implant and subsequent long-term osseointegration. It requires limited micromotion of < 40 μm of the acetabular cup to prevent the formation of fibrocartilaginous membrane and promote adequate membranous bone formation at the bone-implant interface [1]. The initial stability of the acetabular cup in primary THA is gained mainly through press-fitting an implant, with optional augmentation using trans-acetabular screws. The necessity of optional augmentation with trans-acetabular screws remains still controversial [2–8].

The data from the National Inpatient Sample registry in the USA demonstrated a 28.50% increase in patients receiving revision THA from the years 2006 to 2014 [9]. As the demands for THA have been increasing, a 43–70% increase in revision THA frequency from 2014 to 2030 is anticipated [9–13]. Surgeons often encounter a very challenging situation when performing revision THA and are required to make wise decisions within the limited surgery timeframe. Sufficient fixation is often challenging to achieve, especially in revision THAs with large acetabular bone defects [14]. In situations where the initial stability cannot be gained through press-fitting, rigid construct stiffness aided by additional screw insertion is believed to reduce excessive cup-bone interfacial micromotion, supporting bony ingrowth [15]. In the longer term, threads of the acetabular screw also act as an effective structure for the bony ingrowth to occur [16, 17].

In revision surgery with significant segmental acetabular defects, adequate implant selection and fixation methods are critical in determining successful bony ingrowth. Commercially available total hip prosthesis manufacturers generally offer additional multi-hole options of acetabular shells with identical designs for use in revision THAs where screw hole configurations vary from product to product. Apart from acetabular reconstruction cages with flanges or optional metal augments, the configurations provided are diverse from radially symmetrical configuration [18, 19] to holes concentrated in particular directions [20–22] with or without additional rim holes [23]. Still, biomechanical studies regarding the configuration of screw placement are relatively scarce [8, 21, 23].

**Fig. 1:**
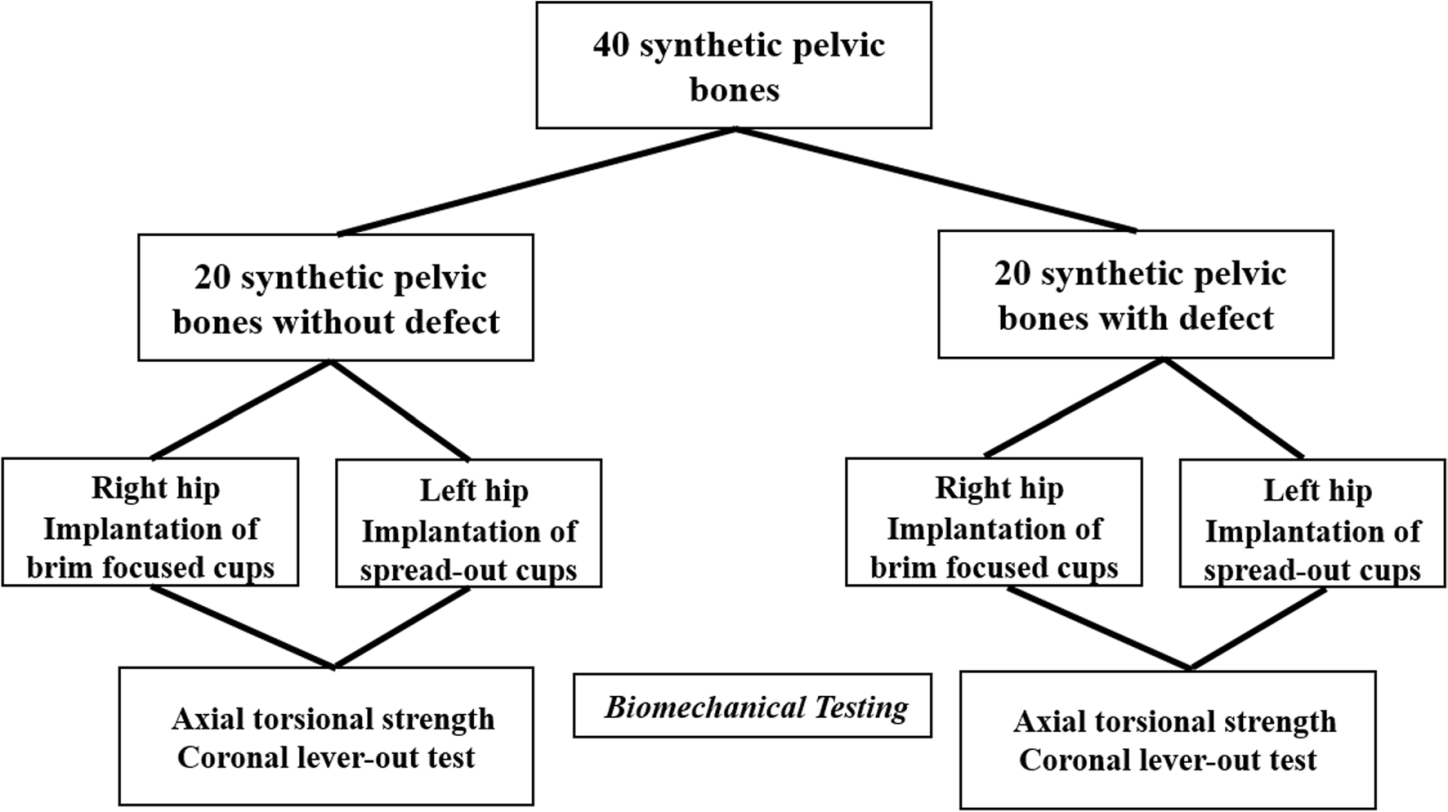
Flowchart of this study

Therefore, this study aims to compare the mechanical stability of the two types of acetabular screw constructs for the fixation of acetabular components: spread-out and pelvic brim-focused configurations. Using pelvic synthetic bone models, the two types of constructs were tested and compared against models with or without artificially created segmental acetabular defects in terms of axial torsional strength and coronal lever-out strength (Fig. 1).

## Materials and methods

### Specimen preparation: synthetic bone models

**Fig. 2:**
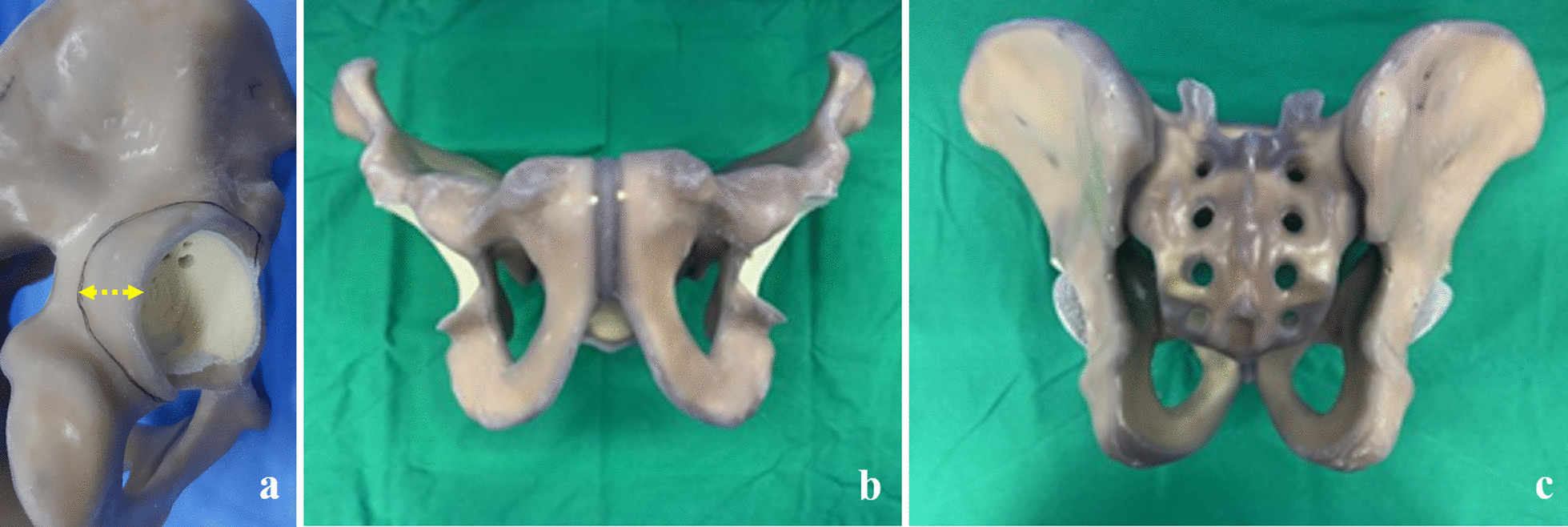
Synthetic bone models of the whole pelvis with bone defects. Among forty synthetic bone specimens, both acetabuli of twenty specimens were cut with an electrical saw to generate posterior segmental defects (**a**), with a maximum width of 20 mm in the posterior 9’o-clock direction (yellow dotted line). The anterior view of the pelvis shows visible posterior bone defects (**b**), and the posterior view with implants inserted is demonstrated with the posterior overhang of the acetabular shells (**c**)

Forty synthetic bone models (Sawbones model 3415–1, Pacific Research Laboratories, Vashon, WA) of the whole pelvis consisting of a short fiber-filled epoxy cortical shell and a 17 pound per cubic feet (PCF) solid rigid polyurethane foam cancellous core were used in this study (Fig. 2). The 4th generation composite synthetic bone model is known to embody the physical properties of human bone in terms of reaming, broaching, and implant fixation. The synthetic bone specimens were divided into two groups: twenty synthetic bone models without acetabular defects and twenty synthetic bone samples with acetabular defects created in the laboratory.

### Specimen preparation: acetabular cup fixation and defect generation

Acetabular reaming was done by a senior high-volume arthroplasty surgeon. The reaming size was raised to 59 mm then an acetabular cup with an outer diameter size of 58 mm was inserted, establishing one-millimeter over-reaming to intentionally exclude press-fitting of the acetabular cup. In this manner, forty acetabular cups were implanted into twenty synthetic bone specimens without any acetabular defects. For the preparation of forty acetabuli in half the samples with acetabular defects, identical curvilinear bone defects were manually created using an oscillating electrical saw (Fig. 2a, b). This segmental defect (Paprosky classification type II) was designed to have a maximum width of 20 mm at the posterior side of the acetabular wall, which ends with 10 mm width defects at 12’ o-clock (superior) and 6` o-clock (inferior) directions (Fig. 2a).

**Fig. 3:**
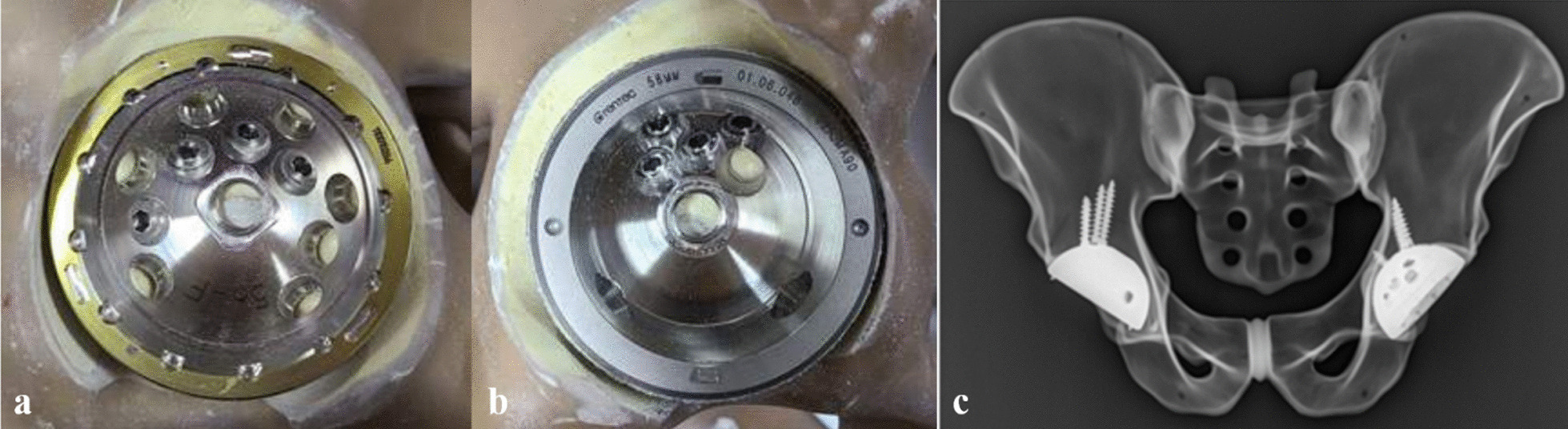
Acetabular cup fixation methods for the preparation for mechanical testing. After creating intentional defects to both acetabuli of the sawbones, four trans-acetabular screws were inserted in the configuration as seen above. Acetabular components containing spread-out configuration screw holes were inserted with screws to the acetabulum (**a**), and acetabular components containing brim-focused configuration screw holes to the left acetabulum (**b**). The resulting screw configuration was confirmed using the image intensifier in the laboratory (**c**)

On each right acetabulum, multi-hole acetabular cups (G7^®^ Acetabular System OsseoTi Multi-hole, Zimmer Biomet, Warsaw, IN, USA) with screw holes in the spread-out configuration were implanted (Fig. 3a) with four conventional acetabular screws from the same manufacturer with following lengths: 20 mm, 30 mm, 30 mm, 30 mm. For each right acetabulum of all 40 synthetic bone specimens, multi-hole acetabular cups (Bencox^®^ Hybrid cup, Corentec, Cheonan, Korea) in which the direction of the screw holes are focused on the superior central areas of the cup or the pelvic brim were fixed using four acetabular screws vice versa (Fig. 3b): 30 mm, 30 mm, 35 mm, 40 mm. The resulting acetabular screw configuration was confirmed under the image intensifier (Fig. 3c). Detailed specifications of the two multi-hole cups used in the study are described in Table 1.

**Table 1:** Characteristics of acetabular cups used in the study

Screw hole configuration	Manufacturer	Product name	Cup shape	Surface coating	Screw holes	Cup size
Brim-focused	Corentec (Cheonan, Korea)	Bencox^®^ hybrid cup	Hemispherical	Plasma porous sprayed Ti	11	58 mm O.D
Spread-out	Zimmer Biomet (Warsaw, IN)	G7^®^ acetabular system multi-hole	Hemispherical	Plasma porous sprayed Ti	5	58 mm O.D

*Ti* Titanium, *O.D.* Outer diameter

### Biomechanical testing: axial torsional strength

Utilizing the mechanical testing machine (MTS 858, MTS system Corp., MN, USA), the coronal lever-out and axial torsion tests were performed to measure the lever-out and torsional load required to displace the fixed implant. Testing was conducted in a controlled environment of 37 °C room temperature and 30% humidity. Axial torsional strength and coronal lever-out tests were done with and without the acetabular defect for each specimen.

**Fig. 4:**
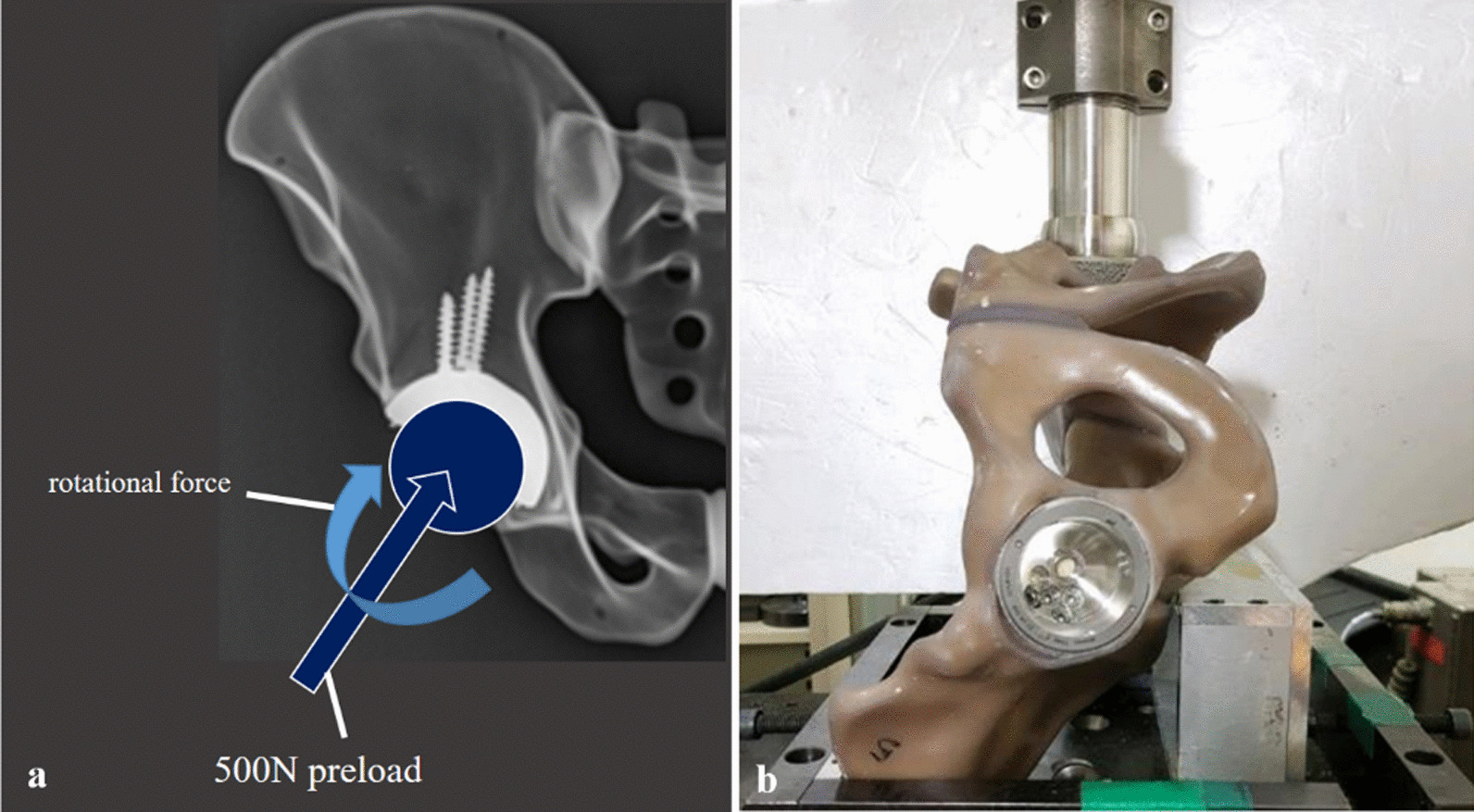
Axial torsional strength test using Instron testing machine. The acetabular cup was fixed, and the rotational force was applied using the mechanical testing machine (MTS 858, MTS system Corp., MN, USA) to record the results into torque–angle curves and establish the axial torsional strength (**a**, **b**)

To perform the axial torsional strength test, specimens were firmly mounted on the testing machine to place the acetabular cup perpendicular to the vertical axis (Fig. 4a, b). 500 N of Compressive load (= pre-load, for structural stabilization) is applied. The test was conducted by applying a torsional load at a speed of 0.038 rad/min (2.18/min). As a result, maximum sustained torque was obtained. The results were recorded into load–displacement or torque–angle curves (Fig. 6a, b). The maximum sustained torque (N m) was determined from the curves for each implant specimen.

### Biomechanical testing: coronal lever-out test

**Fig. 5:**
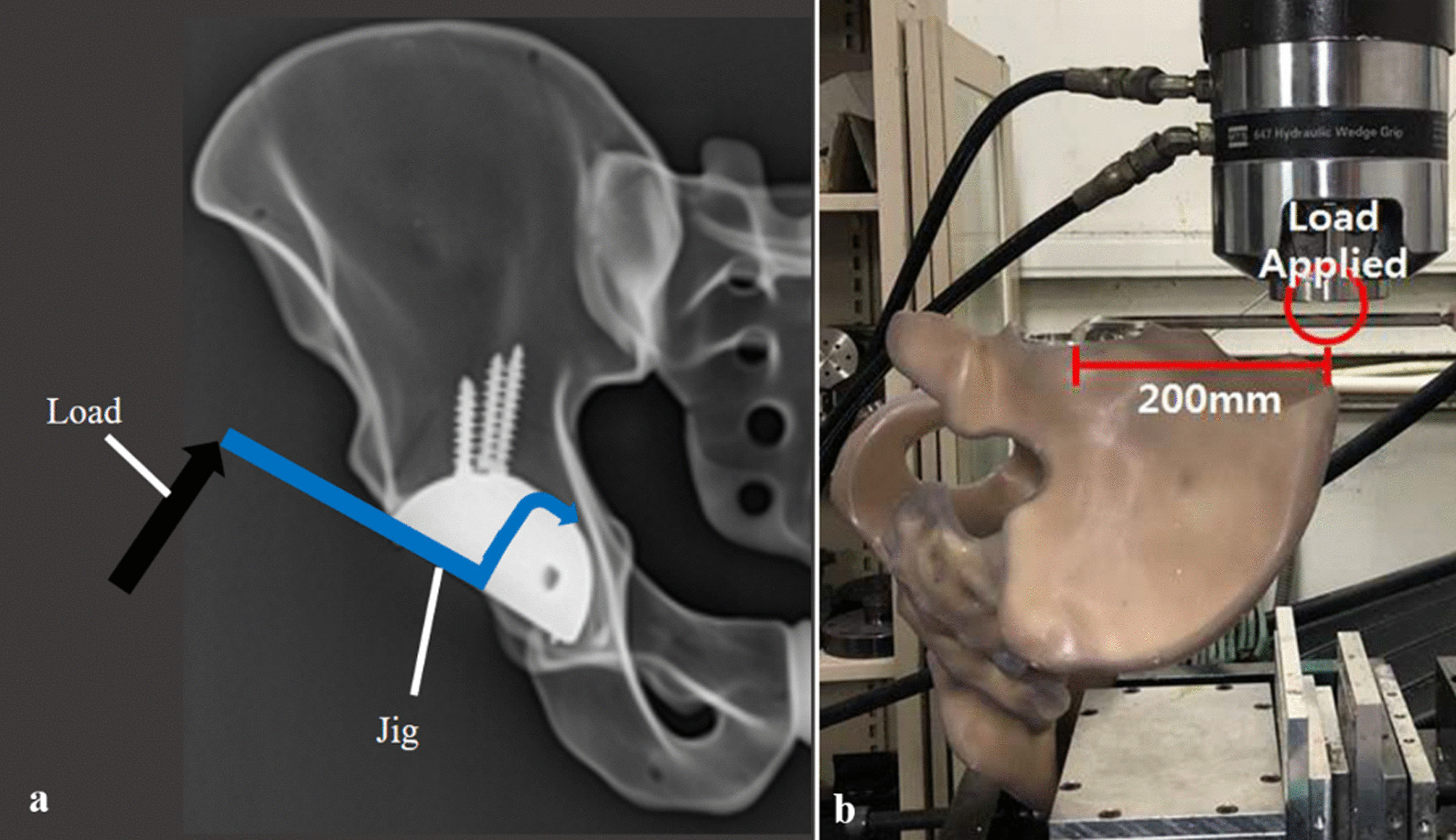
Coronal lever-out test using Instron testing machine. A Jig was connected to the center of an acetabular cup, and the load was applied to a point 200 mm distal from the center to record the results into moment-angular curves and establish the coronal lever-out strength (**a**, **b**)

**Fig. 6:**
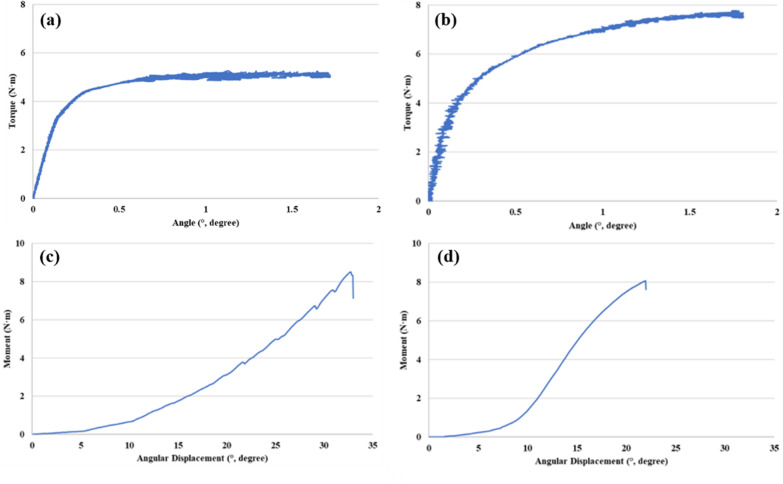
Representative results of the torque–angle curve and moment-angular displacement. To establish the axial torsional strength, torque–angle curves were recorded for each of the brim-focused constructs (**a**) and the spread-out constructs (**b**) which were implanted in acetabuli with the posterior segmental defect. Likewise, to establish the lever-out strength, moment-angular displacement curves were recorded for each of the brim-focused constructs (**c**) and spread-out constructs (**d**)

In an identical environment to the torsional strength test, the coronal lever-out tests were conducted using the mechanical testing machine. The specimen was firmly mounted, and a jig was connected to the center of the fixed implant (Fig. 5a, b). The load was applied at the other side of the jig at a point 200 mm distant from the center of the implant. The degree (*θ*) of displacement was calculated by the arctangent of displacement length over the moment arm, where the moment of force is the product of the moment arm, and the magnitude of the force applied. Variables were recorded into a load–displacement curve and then converted into a moment-angular displacement curve to establish an implant’s lever-out strength (N m) (Fig. 6c, d).

### Statistical analyses

Paired data were gained from each synthetic bone pelvis that contains two acetabuli implanted with a spread-out configuration cup for one acetabulum and a brim-focused configuration cup for the other. Observation data were grouped by defect status, and test types were then tested for normality using the Kolmogorov–Smirnov test and the Shapiro–Wilk test. Paired *T*-tests were used for all groups of continuous variables: the torsional and lever-out strength. *p* values under 0.05 were considered statistically significant. All statistical analyses were conducted using SPSS (version 26.0, SPSS Inc., Chicago, IL, USA).

## Results

Among 40 synthetic bone specimens (80 acetabuli), 38 synthetic bone specimens (76 acetabuli) were adequately tested for recording and eligible for statistical analyses. Two specimens (four acetabuli) were damaged by trial and error, which rendered then unsuitable. One model without defect was cracked during an acetabular cup fixation, and the other specimen was unfit for testing due to excess damage that occurred while creating the defect.

The results of recordings from the tests for the comparison of spread-out and brim-focused acetabular screw configuration in each setting are as in Table 2.

**Table 2:** The results of mechanical machine testing for the axial torsion test and the coronal lever-out test

Groups	Without acetabular defect		*p*	With acetabular defect		*p*
	Spread-out	Brim-focused		Spread-out	Brim-focused	
Average axial torsion strength ± SD (range), N m	26.27 ± 2.12	22.82 ± 1.81	< 0.001^†^	8.23 ± 1.26	6.65 ± 1.58	< 0.001^†^
Average coronal lever-out strength ± SD (range), N m	15.05 ± 1.16	12.92 ± 0.91	0.004^†^	9.90 ± 1.61	10.35 ± 1.69	< 0.001^†^

^†^These *p* values were less than 0.05

The average torsional strengths were significantly higher in the spread-out group over the brim-focused group regardless of the presence of the segmental defect of the acetabulum (*p* < 0.001). But for the lever-out strength, the spread-out group exhibited significantly higher average strength over the brim-focused group for the intact acetabulum (*p* = 0.004); conversely, the brim-focused group with the defects exhibited higher sustained torque (*p* < 0.001).

Both the torsional strength and the lever-out strength drastically declined with the relatively poor fixation power of the acetabular cup. The percent reductions by the presence of acetabular defects were similar for the average torsional strengths of the two groups (68.66% vs. 70.86%). In comparison, the decrease in the average lever-out strength was less significant for the brim-focused group than the spread-out group (19.87% vs. 34.25%) (*p* < 0.001).

## Discussion

Increasing revision THA urges surgeons to often cope with challenging situations and make difficult decisions to achieve the long-term stability of an acetabular component successfully. Nevertheless, there have been promising reports of gaining osseointegration from cementless porous-coated implants for revision THA with an 88–98% 10-year survivorship [24–28]. But it is often difficult to obtain initial stability in resulting revision settings, particularly with acetabular bone defects, where supplemental screw fixations can play an essential role in maximizing the stability and enhancing the contact of the host bone-implant interface [29].

Although it is recommended that same-size or line-to-line reaming with additional screw fixation is adequate for revisional THAs with limited or fragile bone stock [30, 31], sufficient press-fitting is often unachievable. Besides, both generic acetabular components used in this study were manufactured using the same porous titanium plasma spray coating for the surface treatment; differences in the microscopic specification, as well as the number of screw holes, result in a discrepancy in the effective surface area even with the identical outer diameter of 58 mm. Thus, in this study, one-millimeter over-reaming was done prior to screw fixation of all samples to minimize the effect of press-fitting on mechanical cup stability and focus on the comparison of spread-out and brim-focused screw constructs. Even if it can be vaguely thought that the more significant number of effective acetabular screws inserted, the better stability we can gain, clarifying configurations with superior stability in certain conditions would help aid successful revision surgery.

In a biomechanical study, Hsu et al. [20] reported that an acetabular cup with pelvic brim-focused screw hole configuration induces a very stable region at the superior central region, though having little effect on reducing the peak micromotion at the opposite rim edge. In another study, a revision cup with eccentric screw holes provided stability by decreasing the hip rotation center, aiding better biomechanical function [21]. Similarly, by using the Stryker Orthopaedic Modeling and Analytics (SOMA) database [32], Faizan et al. [23] suggested that a modified eccentric jumbo shell with an offset center of rotation exhibit several advantages over generic acetabular shells, which includes providing adjunctive screw holes to the superior rim directed into the posterior column of the pelvis. The acetabular screws in the posterior column or inferior ischium are known to provide better stability than acetabular dome screws alone [24]. But to our knowledge, a direct comparison between two generic acetabular components with different modes of screw hole configuration has yet to be reported.

As observed in the results of this study, mechanical testing for the implants inserted into intact acetabuli demonstrated that the spread-out configuration cup showed significantly better stability over the pelvic brim-focused configuration cup by 15.1% (26.27 ± 2.12 vs. 22.82 ± 1.81, *p* < 0.001) in axial torsional strength and 23.8% (8.23 ± 1.26 vs. 6.65 ± 1.58, *p* < 0.001) in coronal lever-out strength. But surprisingly, the presence of posterior segmental defect exhibited mixed results for the two cups. The spread-out construct was significantly more stable in axial torsional strength by 16.5% (*p* = 0.004), whereas the brim-focused construct was significantly better in coronal lever-out strength by 4.5% (*p* < 0.001). In other words, while a spread-out construct is generally an excellent choice for the intact acetabulum, selecting an acetabular cup with a brim-focused screw hole configuration might become a feasible option when a substantial posterior segmental bone defect is present. Likewise, the percent reduction in average coronal lever-out strength (19.87% vs. 34.25%) by the presence of the defect was also reduced in the brim-focused group.

In this study, the spread-out configuration of acetabular screw placement showed superior stability than the pelvic brim-focused configuration in the absence of segmental bone defects. However, when planning revision THA with significant segmental acetabular defects or insufficient bone stock, it would be reasonable to consider using acetabular cups with brim-focused screw hole configuration rather than adhering to the use of cups with spread-out configuration. Nevertheless, buttress graftings such as adjunctive metal buttress augment or bone block allografting for the bone deficit should be considered first to increase the chances of successful surgery.

The limitation of this study is that it is an in vitro biomechanical study using synthetic bone model samples of a limited number of samples. Second, the lengths of the four acetabular screws used to fix the components differ between the two groups, which would considerably affect the discrepancies between the initial stability. This is because when the same length was used, the screw pierced the cortex. The choice of screw length was to insert the most extended screw within the limit of not penetrating the cortex. Nevertheless, this study is not to clarify the effects of the configuration of constructs in the same condition but to compare the mechanical stability of suitable constructs for each generic acetabular shell. Effects of the number of screws, their length, and directions would be dealt with in future studies using computational analysis utilizing the finite element models. Additional biomechanical and cadaveric investigations are required to examine the impacts that vary with the size and location of the bone defects, as well as to compare alternative shell designs that feature an eccentric configuration or supplementary screw holes in the superior rim.

## Conclusion

In this biomechanical study, for synthetic bone models with intact acetabuli, constructs of multi-hole acetabular cups with the spread-out screw holes configuration exhibited statistically better axial torsional strength and coronal lever-out strength over the pelvic brim-focused constructs. In the presence of posterior segmental bone defects, the spread-out constructs demonstrated statistically better tolerance to axial torsional strength. However, the brim-focused constructs showed higher lever-out strength than the spread-out constructs. Therefore, in planning revision THA in a hip with significant acetabular segmental bone defects, adjunctive brim-focused screws may aid initial mechanical stability regarding lever-out strength, in addition to essential torsional stability gained by the spread-out configuration of acetabular screws.

## Data Availability

The datasets analyzed during the current study are available from the corresponding author on reasonable request.
